# Separation of responsive and unresponsive patients under clinical conditions: comparison of symbolic transfer entropy and permutation entropy

**DOI:** 10.1007/s10877-023-01046-w

**Published:** 2023-07-12

**Authors:** Robert Zanner, Sebastian Berger, Natalie Schröder, Matthias Kreuzer, Gerhard Schneider

**Affiliations:** 1 Department of Anesthesiology, HELIOS University Clinic Wuppertal, Witten/Herdecke University, Heusnerstr. 40, 42283 Wuppertal, Germany; 2 Department of Anesthesiology and Intensive Care, School of Medicine, Technical University of Munich, Ismaninger Str. 22, 81675 Munich, Germany; 3 Klinikum Fünfseenland, Robert-Koch-Allee 6, 82131 Gauting, Germany

**Keywords:** EEG monitoring, General anesthesia, Frontal-parietal connectivity, Symbolic transfer entropy, Permutation entropy

## Abstract

Electroencephalogram (EEG)-based monitoring during general anesthesia may help prevent harmful effects of high or low doses of general anesthetics. There is currently no convincing evidence in this regard for the proprietary algorithms of commercially available monitors. The purpose of this study was to investigate whether a more mechanism-based parameter of EEG analysis (symbolic transfer entropy, STE) can separate responsive from unresponsive patients better than a strictly probabilistic parameter (permutation entropy, PE) under clinical conditions. In this prospective single-center study, the EEG of 60 surgical ASA I–III patients was recorded perioperatively. During induction of and emergence from anesthesia, patients were asked to squeeze the investigators’ hand every 15s. Time of loss of responsiveness (LoR) during induction and return of responsiveness (RoR) during emergence from anesthesia were registered. PE and STE were calculated at −15s and +30s of LoR and RoR and their ability to separate responsive from unresponsive patients was evaluated using accuracy statistics. 56 patients were included in the final analysis. STE and PE values decreased during anesthesia induction and increased during emergence. Intra-individual consistency was higher during induction than during emergence. Accuracy values during LoR and RoR were 0.71 (0.62–0.79) and 0.60 (0.51–0.69), respectively for STE and 0.74 (0.66–0.82) and 0.62 (0.53–0.71), respectively for PE. For the combination of LoR and RoR, values were 0.65 (0.59–0.71) for STE and 0.68 (0.62–0.74) for PE. The ability to differentiate between the clinical status of (un)responsiveness did not significantly differ between STE and PE at any time. Mechanism-based EEG analysis did not improve differentiation of responsive from unresponsive patients compared to the probabilistic PE.

*Trial registration*: German Clinical Trials Register ID: DRKS00030562, November 4, 2022, retrospectively registered.

## Introduction

Monitoring the effects of anesthetic drugs on the brain using EEG derived parameters has been increasingly adopted into clinical practice in the recent past. For this purpose, several monitors are commercially available, i.e., the Bispectral Index (BIS) Monitor (Medtronic, Meerbusch, Germany), the Narcotrend (Narcotrend Gruppe, Hannover, Germany), the Entropy Module (GE Healthcare, Solingen, Germany) or the Conox (Fresenius Kabi, Bad Homburg, Germany). All these monitors register frontal EEG and *M. facialis* electromyogram (EMG) to calculate an index using proprietary algorithms. The calculated indices usually range between 100 (fully awake) and 0 (total suppression of cortical function) and pretend to reflect the level of anesthesia.

The use of these monitors may reduce complications resulting from inappropriate dosing of anesthetic drugs. However, the convincing proof in a prospective, randomized trial is still lacking in the current literature. For example, the BIS—probably the most examined of these monitors—was not found to reduce the incidence of awareness [1] in a large study. Considering delirium, conflicting results have been published [2, 3]. Several possible limitations of these monitors have been identified in the past, i.e., a time delay of index calculation [4–6], the effect of age-dependent changes of the EEG on index calculation [7–9] or the focus on power spectrum parameters in the calculation algorithms [10].

Anesthetic-induced loss of consciousness has recently been linked to changes in the complexity present in frontal-parietal information transfer using electrophysiology and functional imaging. Several studies suggest that unconsciousness induced by anesthetic drugs is associated with a decrease in connectivity of frontal-parietal networks as assessed by fMRI [11–14] whereas primary sensory networks appear to be unaffected [15, 16]. We hypothetized that EEG analysis which relates to the presumed mechanism of anesthetic-induced unconsciousness, i.e., a change in fronto-posterior cortical connectivity, may overcome the limitations of the probabilistic approach of commercial monitors and improve anesthetic drug monitoring during surgery under general anesthesia.

We therefore recorded perioperative EEGs of 60 surgical patients and compared the ability of STE—representing a more mechanism-based approach—and permutation entropy (PE)—representing a strictly probabilistic approach—in differentiating responsive from unresponsive patients at loss and return of responsiveness. Our setup for EEG recording and analysis was chosen in a way suitable for routine use in the operating theater.

It has to be noted, that unresponsiveness does not equal unconsciousness. However, unconsciousness is not a prerequisite in order to prevent a patient from experiencing surgery. It is more important, that the patient is disconnected from the environment [17]. Assessing a patient’s response to a specific command (so-called goal-directed responsiveness) is an acceptable clinical method to identify patients that are conscious and connected, although they seem unconscious at first sight. E.g., using this methodology, recent studies showed that connected consciousness after tracheal intubation occurred in 5% of patients [18], in young adults the incidence was even higher at 11% [19].

## Materials and methods

### Patient recruitment

With approval of the local ethics committee, 60 adult patients (inclusion rate 92.3% of screened patients) undergoing elective non-cardiac and non-neurosurgical procedures under general anesthesia at HELIOS University Hospital Wuppertal, Germany, gave informed written consent to be included in the study. Patients were recruited during a 5-month period from August to December 2015. Exclusion criteria were American Society of Anesthesiologists (ASA) physical status higher than IV, a history of psychiatric or neurologic diseases, known drug abuse, medication or surgery affecting the central nervous system, pregnancy, or an indication for rapid-sequence induction. No randomization procedure was applied.

### Study design and EEG recording

During preparation for anesthesia, EEG electrodes were placed at Fp1, Fp2, P3, P4, CPz (common reference), and AFz (ground) positions using a MultiCap Flat EEG system cap (GVB-Gelimed. Bad Segeberg, Germany). For EEG recording, the NIM-ECLIPSE®-system (Medtronic, Jacksonville, USA) was used. EEG recording was started before anesthesia and continued until patients were transferred to the postanesthesia care unit.

After recording a baseline (no manipulation on the patient, eyes open or closed to the discretion of the patient) for 1–2 min, an opioid (sufentanil or remifentanil) was applied. When patients reported clinical signs of opioid effect (e.g. light-headedness, warm feeling, etc.), induction was started with 20 mg boluses of propofol every 15s (= induction of anesthesia). At the same time, patients were asked to squeeze the investigator’s hand. The time point, when patients did not react to repeated verbal command was noted as loss of responsiveness (LoR). After securing the airway, anesthesia was maintained throughout surgery with either sevoflurane or propofol at the discretion of the attending anesthetist.

At the end of surgery, starting 3 min after anesthetics were discontinued (= emergence from anesthesia), patients were again requested to squeeze the investigator’s hand. In the beginning, every minute and when spontaneous ventilation recurred every 15s. The time point, when the patient first obeyed the verbal command was noted as return of responsiveness (RoR).

### Ordinal entropy measures

#### Permutation entropy (PE)

PE is a nonlinear parameter that quantifies the regularity structure of the neighboring order of signal values in order to reflect the information content of the signal [20]. It is very robust against artefacts and easy to calculate [21]. Applied to the EEG, PE has been shown to reliably separate consciousness from unconsciousness [22].

#### Symbolic transfer entropy (STE)

STE was introduced by Staniek and Lehnertz in 2008 [23]. With PE, it shares the analysis of amplitude orders instead of absolute values. In contrast to PE, STE not only reflects information content of one EEG channel, but also gives information about the directed mutual interaction between a pair of EEG electrodes.

Although several studies by independent laboratories found decreased information transfer in frontal to parietal direction using imaging [15, 16], electrophysiology [11, 13, 24, 25], and both [12, 14], these results have been challenged by some studies using different methodology [26]. To account for these different findings, we investigated STE in both, frontal to parietal and parietal to frontal directions. Furthermore, we added up STE values from both directions to create an undirected value that represents the total information content.

### EEG analysis

To evaluate STE under online patient monitoring conditions, we designed all EEG processing steps to be *causal* in the sense of systems theory, such that the system output $\varvec{y}\left(t\right)$ at time $t$ does not depend on input data $\varvec{x}\left(t+ \varDelta t\right)$ with $\varDelta t>0$. In other words, the system may not look into the future.

All computations were performed using Matlab 2017b (MathWorks, Natick, MA, USA).

#### EEG preprocessing

The EEG, originally recorded at 250 Hz sampling rate, was limited to the frequency range 0–30 Hz by adequate filtering and then re-sampled at 200 Hz. To avoid phase distortions while still maintaining system causality, the following approach was used: EEG signals were first upsampled to 1000 Hz by zero-padding. A 1000th order finite impulse response (FIR) filter was then applied for band-limiting and anti-aliasing. In a last step, downsampling to 200 Hz was performed by selecting every fifth sample of the filtered signal. This procedure provides perfect phase coherence at the expense of introducing a constant time delay of 500 ms.

#### Ordinal pattern extraction and data segmentation

For each patient, each channel of EEG was encoded into a sequence of ordinal patterns (embedding dimension $m=5$, time lag $\tau =1)$ using Matlab and the *libordpat* library [27]. The pattern sequences were then segmented using a sliding window of 30s length and a time shift of 1s, yielding a data block of 5960 patterns × 4 channels per position of the sliding window. Each data block was assigned the time stamp of the rightmost of its underlying EEG samples.

#### Entropy estimation

The STE of an ordered pair of EEG epochs is the transfer entropy (TE) of their pair of ordinal pattern sequences. Schreiber [28] defined the TE from one discrete stochastic process $X$ to another such process $Y$ as the divergence from a generalized Markov property, which leads to ${T}_{X\to Y}=\text{H}\left({Y}_{t+\delta }| {Y}_{t}\right)- \text{H}\left({Y}_{t+\delta }| {X}_{t}, {Y}_{t}\right)$. Reformulation in terms of ${T}_{X\to Y}=\text{H}\left({{Y}_{t}, Y}_{t+\delta }\right)- \text{H}\left({Y}_{t}\right)- \text{H}\left({{{X}_{t, }Y}_{t}, Y}_{t+\delta }\right)+ \text{H}\left({X}_{t}, {Y}_{t}\right)$ provides a recipe for estimating TE from *realizations* of processes, that is, from sequences of measurement values: the probability distributions underlying the four entropies are obtainable by counting the relative frequencies of (tuples of) values.

In this manner, we computed a set of STE estimates from each of the data blocks described in Sect. 2.4.2. In particular, STE was estimated for the ordinal pattern sequences of the fronto-parietal channel pairs (Fp1 → P3), (Fp1 → P4), (Fp2 → P3), (Fp2 → P4), as well as the parieto-frontal channel pairs (P3 → Fp1), (P3 → Fp2), (P4 → Fp1), (P4 → Fp2). The couplings were assessed for six different transfer delays $\delta =7, 8, \dots , 12$, that is, for 35ms, 40ms, …, 60ms.

Thus, we computed a set of 48 STE values per each data block. To obtain a single score that represents the average coupling strength between frontal and parietal regions, their mean value was calculated similar to the procedure in previous studies using STE [12–14], and this overall value was timestamped in accordance with the underlying data block.

Analogously, we obtained the averaged PE of the frontal EEG channels Fp1 and Fp2 for each data block. (Notice that the estimation of ${T}_{X\to Y}$ yields $\text{H}\left(Y\right)$ as a side product).

As mentioned earlier, both approaches have been used in the context of EEG-based anesthesia monitoring [22, 29].

### Statistical analysis

**Fig. 1:**
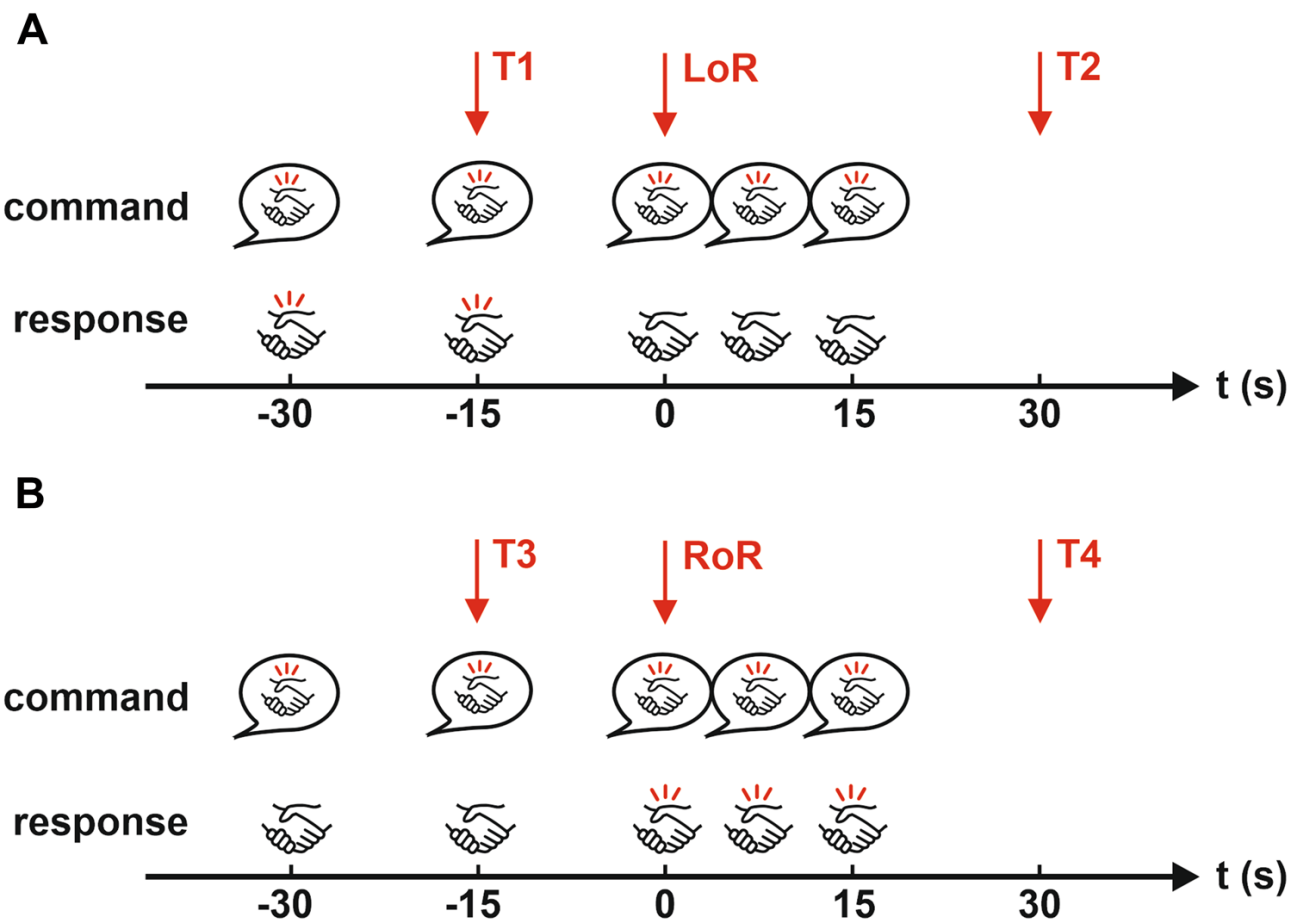
Time points (T) for analysis of entropy parameter performance during **A** induction of and **B** emergence from anesthesia. Performance was analyzed 15s before loss (LoR; T1) and return of responsiveness (RoR; T3) and 30s after LoR (T2) and RoR (T4). Verbal commands are depicted in speech bubbles, the patient’s response below

We analyzed the performance of PE and STE at four distinct time points (Fig. 1): LoR −15s (T1), LoR +30s (T2), RoR −15s (T3), and RoR +30s (T4). This selection is motivated as follows: During the induction of general anesthesia, LoR −15s is the latest point in time that does not contain EEG from the anesthetized patient state, because the patient had still squeezed the investigator’s hand at LoR −15s. Furthermore, LoR +30s is the earliest point in time that does not contain EEG from the wakeful patient state, because the patient had not squeezed the investigator’s hand at LoR and the analysis window is 30s long. The same principle applies to the selection of RoR −15s and RoR +30s.

To analyze and compare the classification performances of the EEG parameters during induction of and recovery from general anesthesia, we used the accuracy statistic. Accuracy can be calculated as follows:

$$\frac{{t}_{p} + {t}_{n} }{{t}_{p}+{f}_{p}+{t}_{n}+{f}_{n} }$$

where *t*_*p/n*_ stands for true positive/negative and f_p/n_ for false positive/negative. In our case, the calculated accuracy resembles the probability of a patient’s state of responsiveness being correctly classified by the examined parameter.

For both PE and STE, we dichotomized the parameter values by selecting the threshold that maximizes the accuracy for LoR −15s vs. LoR +30s. We used this threshold for all accuracy analyses: any STE or PE value higher than the threshold was considered “positive for wakefulness”, and “negative for wakefulness” otherwise. For pairwise comparison between accuracies, we computed their differences. For absolute accuracies as well as their differences, we applied 10,000-fold bootstrap re-sampling to compute 95% confidence intervals.

## Results

### Patient data

We had to exclude 4 out of 60 study patients from the analysis due to incomplete EEG recordings after RoR. The patients’ demographics are summarized in Table 1.

**Table 1:** Characteristics and types of surgery for n = 56 included study patients

Age	(Ø±std. dev. [range]) y	50.4±19.3 [20–81]
Weight	(Ø±std. dev. [range]) kg	89±20 [52–150]
Height	(Ø±std. dev. [range]) cm	178±8 [157–198]
Male sex	(%/n)	78.6%/44
ASA category I	(%/n)	57.1%/32
II	(%/n)	37.5%/21
III	(%/n)	5.4%/3
IV	(%/n)	0%/0
Type of surgery	urology (%/n)	35.7%/20
	trauma (%/n)	23.2%/13
	abdominal (%/n)	17.9/10
	plastic (%/n)	17.9/10
	dermatology (%/n)	5.4%/3

In all patients, anesthesia was induced with propofol. 15 patients received total intravenous anesthesia, whereas in 41 patients, anesthesia was maintained using a volatile agent. Duration of anesthesia from LoR to RoR was 94±63 min. No patient reported awareness with recall as assessed with the Brice questionnaire [30].

### Qualitative behavior of STE and PE

**Fig. 2:**
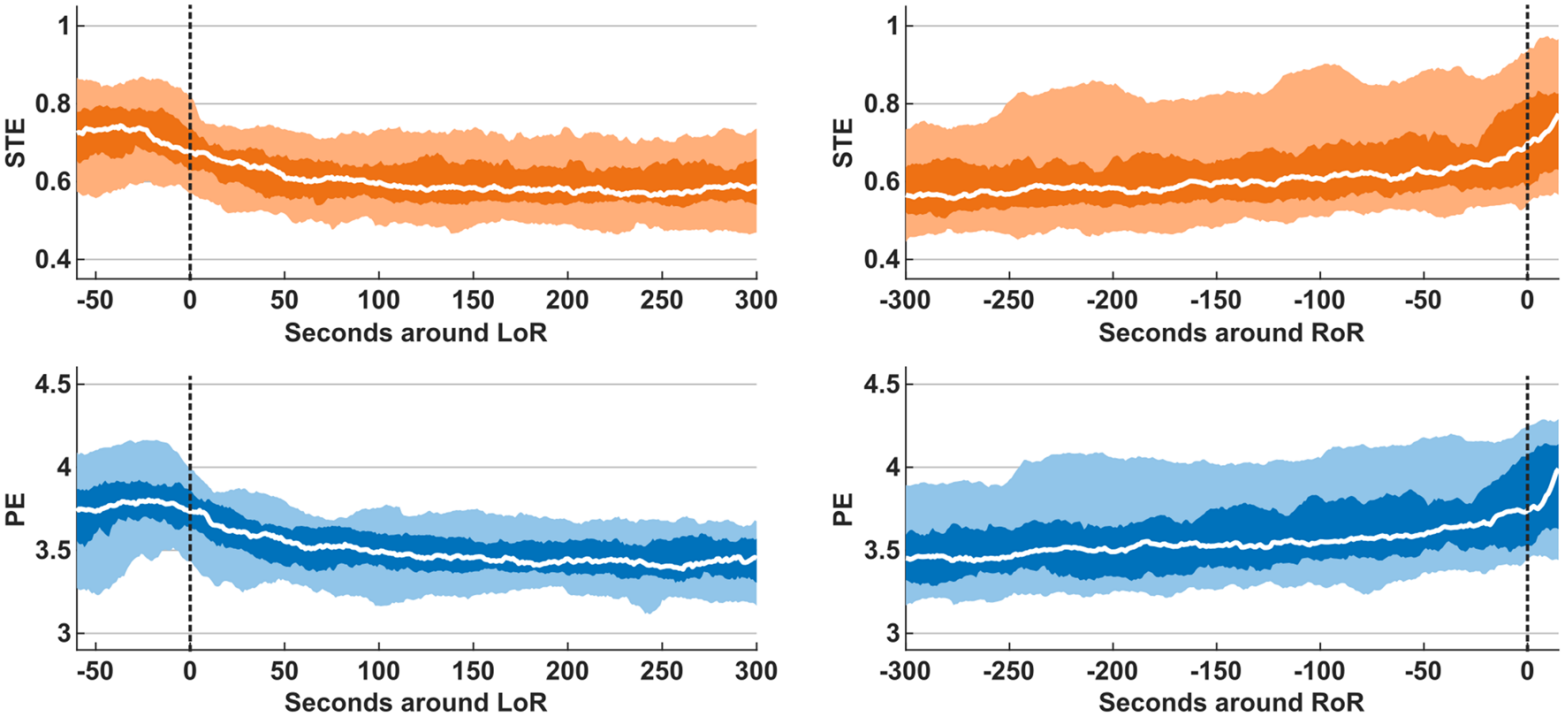
Trend behavior of symbolic transfer entropy (STE) and permutation entropy (PE) scores around loss of responsiveness (LoR) and return of responsiveness (RoR). The medians (white line), 25th to 75th percentiles (dark shaded area), and 5th to 95th percentiles (light shaded area) are depicted for the EEG recordings of *n* = 56 patients. LoR and RoR are depicted as dotted lines

Figure 2 depicts averaged curves for STE and PE scores during induction of and emergence from general anesthesia. Both, STE and PE demonstrated qualitatively comparable behavior. We observed a decrease of STE and PE during induction of general anesthesia, as well as an increase of STE and PE during emergence. STE and PE scores showed higher intra-individual consistency during induction than during emergence. In particular, entropy scores undulated around a stable level before LoR, consistently decreased during the transition phase, and stabilized at a lower value range approximately one minute after LoR. By contrast, both STE and PE increased more slowly during emergence from anesthesia and demonstrated higher intra-individual variation. Furthermore, we observed higher maximum entropy scores after RoR than before LoR.

### STE and PE around LoR and RoR

**Fig. 3:**
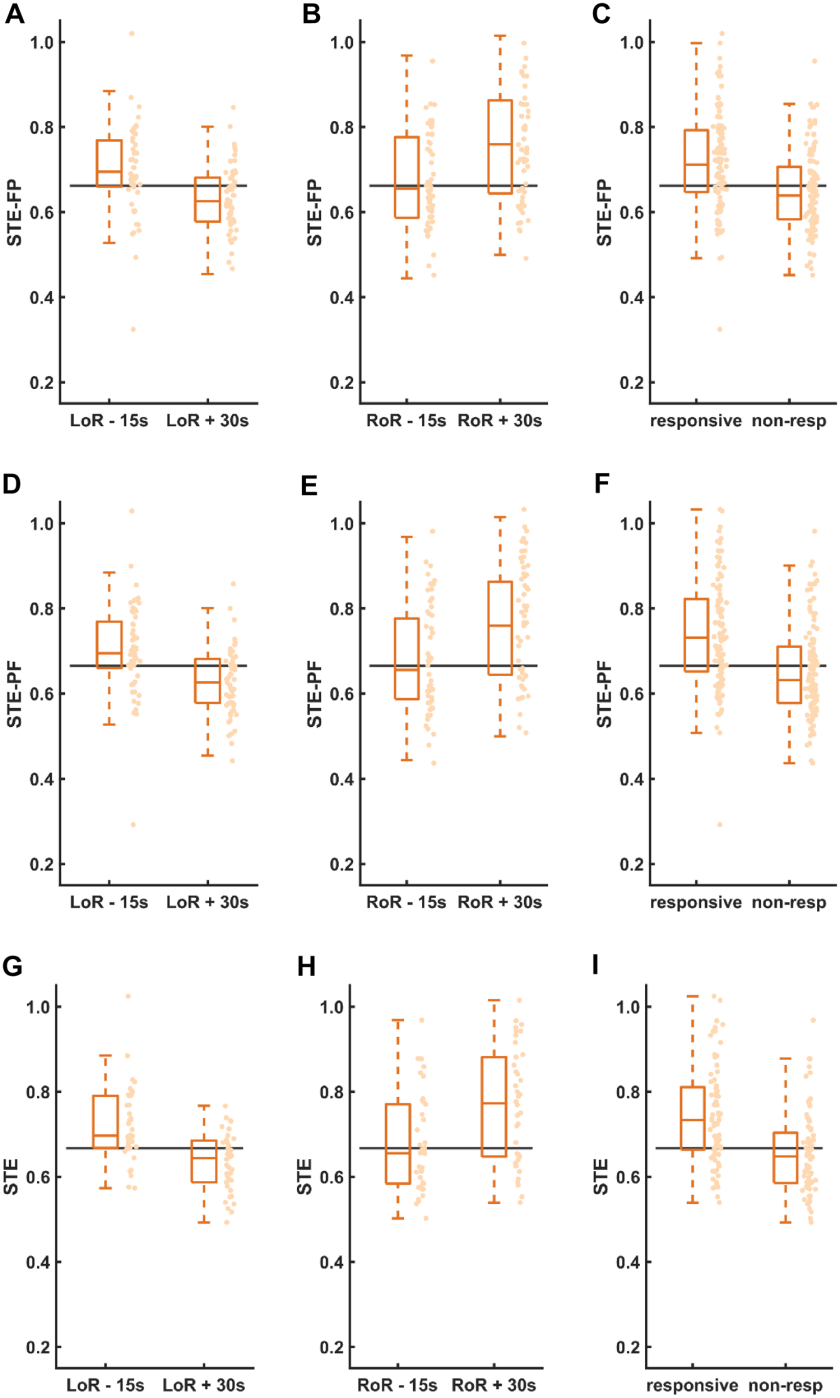
Symbolic transfer entropy (STE) scores for n = 56 patients calculated for the frontal-parietal direction (top), the parietal-frontal direction (middle) and the undirected combination of both (bottom), sampled 15s before and 30s after loss (**A**, **D**, **G**) and return (**B**, **E**, **H**) of responsiveness (LoR, RoR). **C**, **F**, **I** STE scores of responsive vs. non-responsive patients at all time points. Boxes represent the median and 25th to 75th percentiles, whiskers represent the 5th to 95th percentiles. The optimal LoR classification threshold is plotted as a horizontal line

**Fig. 4:**
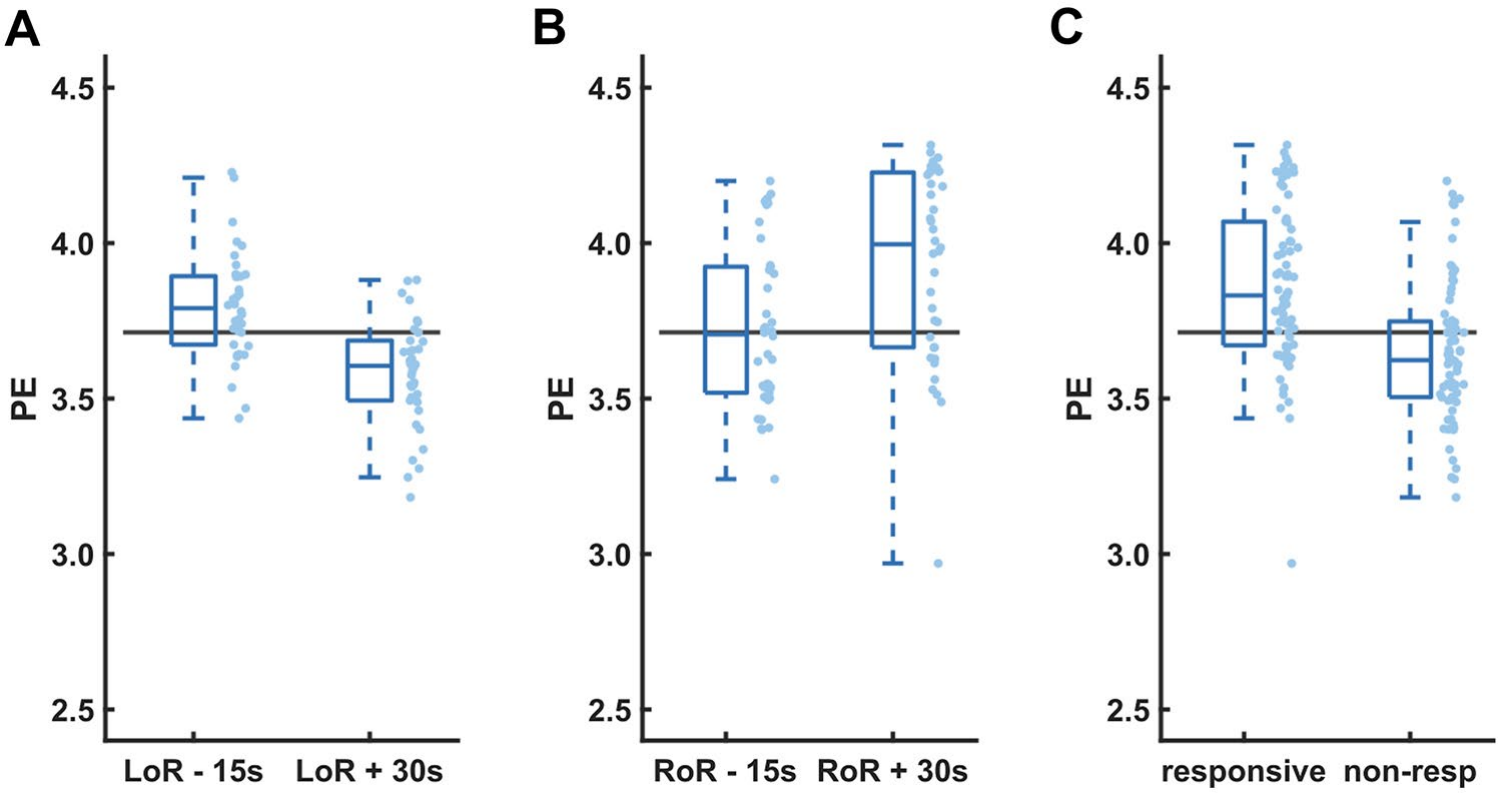
Permutation entropy (PE) scores for *n* = 56 patients, sampled 15s before and 30s after loss (**A**) and return (**B**) of responsiveness (LoR, RoR). **C** PE scores of responsive vs. non-responsive patients at all time points. Boxes represent the median and 25th to 75th percentiles, whiskers represent the 5th to 95th percentiles. The optimal LoR classification threshold is plotted as a horizontal line

**Fig. 5:**
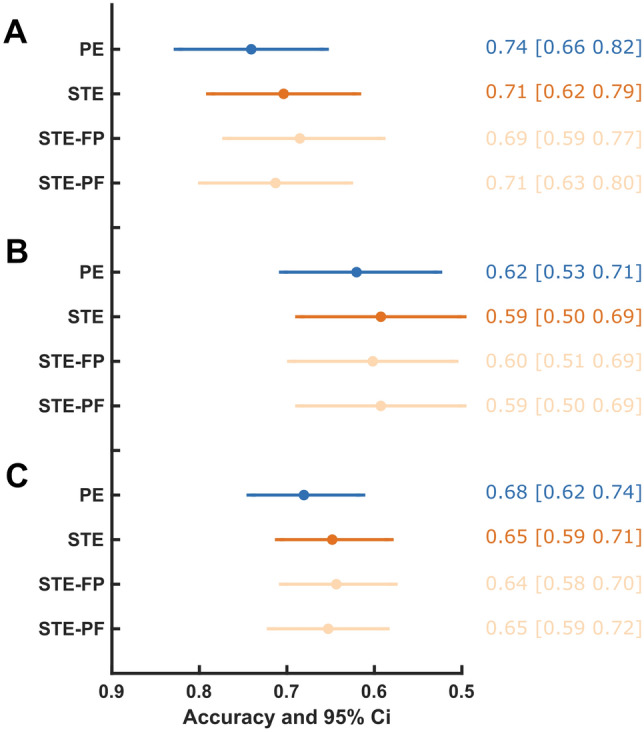
Summary of accuracy statistics with 95% confidence intervals for loss (**A**) and return (**B**) of responsiveness as well as the combination of both (**C**). For all situations there is a considerable overlap in the performance of the parameters with permutation entropy (PE) having the highest accuracy in all situations.; STE: symbolic transfer entropy

Figures 3 and 4 visualize the distributions of STE and PE scores, respectively, at four distinct time points per patient—LoR −15s, LoR +30s, RoR −15s, and RoR +30s—and the combinations of LoR −15s and RoR +30s as well as LoR +30s and RoR −15s. From those samples, we assessed the accuracy of STE and PE when used as binary classifiers for patient responsiveness (Fig. 5). Table 2 shows the accuracy comparison of the different STE parameters with PE.

**Table 2:** Accuracy comparison of symbolic transfer entropy (STE) with permutation entropy for *n* = 56 patients, sampled 15s before and 30s after loss and return of responsiveness (LoR, RoR)

	STE-FP accuracy	STE-PF accuracy	STE-accuracy
LoR	0.06 (−0.06–0.18)	0.03 (−0.09–0.15)	0.04 (−0.08–0.15)
RoR	0.02 (−0.11–0.15)	0.03 (−0.10–0.15)	0.2 (−0.11–0.14)
LoR and RoR	0.04 (−0.06–0.12)	0.03 (−0.06–0.12)	0.03 (−0.06–0.11)

Confidence intervals: 95% confidence level, 10,000-fold bootstrap resampling

## Discussion

According to the global workspace theory, information transfer between anterior and posterior areas of the cortex is crucial for the state of consciousness. STE is a method to quantify the efficiency of this transfer based on EEG signals derived from anterior and posterior areas of the cortex, whereas PE is calculated from an EEG signal derived from the anterior area of the cortex only. The aim of our study was to investigate a possible benefit of a mechanistic-based approach, i.e., STE, to monitor the patient’s anesthetic level during the transitions into and out of unresponsiveness. With our results we could not show that benefit. Both parameters investigated, the multi-channel derived STE and the one-channel based approach PE, were able to distinguish the EEG of a responsive patient from an unresponsive one to some degree. The accuracy analysis including confidence intervals revealed the considerable overlap in performance to distinguish between responsiveness and unresponsiveness. Because of this overlap and the PeEn having the better performance values, we conclude that using STE does not lead to a better separation between these states.

Interestingly, the direction of STE calculation did not affect the determined accuracies to a relevant extent. This finding is in contrast to data published by our own [12, 14] and other research groups [11, 13] and adds more doubts regarding the suitability of STE as a parameter for investigating directional connectivity.

In general, we could describe accuracies around 0.7 for the LoR and around 0.6 for the RoR for both parameters, meaning they correctly categorized 60–70% of the patients. Compared to commercially available monitors, that have been investigated in regard to their ability to separate responsive from non-responsive subjects in a clinical setting, this is a good result. When hand squeezing was used to discriminate awake from unconscious patients, the Narcotrend® monitor showed a prediction probability (P_k_) of 0.5, implying that the performance of this monitor was equal to rolling the dice [31]. Kaskinoro and coworkers [32] investigated the performance of BIS and Spectral Entropy (Entropy Module, GE Healthcare) in healthy male volunteers anesthetized with propofol, sevoflurane or dexmedetomidine. Eye opening upon verbal command was used to discriminate wake and unconscious subjects. In their setting, the authors calculated P_k_ values ranging from 0.52 to 0.73 depending on the used anesthetic and monitor. Due to high inter-individual variation, they concluded that none of the tested monitors was able to differentiate consciousness from unconsciousness.

Both, PE and STE showed a better performance during LoR than during RoR. Similarly, the commercially available qCon monitor (Quantium Medical, Barcelona, Spain) showed a better discrimination during LoR when recorded EEGs from awake and anesthetized patients were replayed [33]. The reasons therefore may be manifold. The trajectory into anesthesia during LoR is rather steep, causing a strong change in the EEG in a shorter time than during RoR. Hence, the contrast is stronger and STE and PE may show higher accuracy. The EEG behavior during RoR may be substance-specific [34] and hence add to the heterogeneity of emergence EEG trajectories that have been described earlier [35–38]. The more heterogeneous EEG during RoR seems to be the reason for the wider STE and PE spread during RoR as depicted in the wider boxes of Figs. 3B, E, H and 4B in contrast to the boxes at LoR. The threshold values for the highest accuracy were higher for RoR than LoR. This could be due to the fact that during emergence and around the RoR the patient seems to be less calm than during anesthesia induction. This could lead to a stronger power of higher frequencies, i.e., a faster EEG which in turn would cause PE and STE to increase.

On the other hand, background brain status at RoR differs from LoR. With low concentration of anesthetics in the brain at RoR, a higher degree of cortico-cortical connectedness may be required to produce executive consciousness.

PE was described to be a suitable parameter for anesthesia monitoring [29]. This also includes a good performance in tracking state transitions [22]. STE was established to evaluate the change in information transfer between frontal and posterior cortical regions during loss and return of responsiveness [11]. Hence, the reduction in information flow as assessed with STE was considered a potential mechanism of losing responsiveness. The same STE features have been described for several GABAergic and non-GABAergic drugs [12–14]. The question in this work was if the inclusion of more, i.e., fronto-parietal EEG information may help to improve the performance of tracking LoR and RoR in a common clinical setting. In our patients, this was not the case. The single channel PE, applied to the frontal EEG typically used for patient monitoring did not show weaker results than STE. For a standardized patient monitoring this implies that additional information regarding fronto-parietal information transfer is not superior to the PE approach, which is easier to apply by only using a single channel frontal EEG and computationally less demanding.

Originally, we had expected that using STE would lead to better results than using PE, because STE analysis reflects changes of the fronto-parietal information transfer, i.e., the suggested mechanism of anesthesia-induced unconsciousness. The breakdown of fronto-posterior connectivity has been suggested as a mechanism underlying anesthesia-induced unconsciousness [12–15]. Recently, this concept has been challenged by studies which found increased functional connectivity during anesthesia. In macaques, long range fronto-parietal cortico-cortical connectivity in the oculomotor circuit was increased. In contrast to previous approaches, this study was focused on the oculomotor circuit with a defined anatomical connection [39]. This underlines the need for differentiated analysis of fronto-parietal connectivity, because the general approach may also include circuits with an increase of connectivity.

But even beyond anatomically connected areas, several methods also suggested an increased functional connectivity [40, 41]. It must be kept in mind that based on the applied analysis method, increased connectivity may not necessarily mean an increased information flow, but may also point towards a reduction of information flow, related to a reduction of complexity or isolation of the analyzed circuit from other areas.

This supports the view that consciousness is related to a balance of cortical dynamics and functional connectivity [42].

These limitations may at least in part explain, why a more mechanism-based approach, STE, does not necessarily need to produce better results than PE. In summary, both approaches allow a good separation between levels of consciousness. STE may more closely reflect the process of information transfer per se (with all limitations of mixed summary effects of increased/decreased connectivity). PE may be a better reflection of the (local) result of altered connectivity.

With potentially opposite changes of functional connectivity in different systems and sub-structures (e.g. functional vs. anatomical connected), analysis of the process itself (STE) may not lead to an unambiguous result. Our results suggest that it may be more important to capture the (locoregional) result of changes in connectivity. This may be more precisely reflected by a regional summarizing parameter, reflecting results of this change. Therefore, PE may be also in theory more suitable than STE, and in daily clinical routine it is also easier to capture, as a single EEG channel can be used for analysis, which makes its application much easier.

When interpreting our data, some limitations have to be kept in mind. In our accuracy analysis, we selected threshold values for dichotomization to maximize accuracy for PE and STE. These values are derived from and valid for our data, but they might not be valid for other datasets.

A further limitation of our study is, that it was not designed to test for possible differences in the performance of PE and STE in subgroups, i.e. balanced anesthesia versus total intravenous anesthesia or male versus female sex. Our study population, showed an imbalance towards male participants (78.6%) and balanced anesthesia (73.2%) so that a post-hoc analysis will not yield valid results due to the low number of female patients and patients that received TIVA. Future studies are needed to investigate these questions. Of course in case of a negative result there is always a possibility of a type II error. Therefore, we state that STE is not performing better than PE, and a type II error neglecting the significantly worse performance of STE would not distort our conclusion.

## Conclusions

In summary, we found that in our clinical setting both examined parameters, PE and STE, distinguished responsive from unresponsive patients to some degree without one parameter being statistically significant superior over the other. Therefore, adding information on frontal-parietal information transfer using STE seems to have no beneficial effect on EEG-based anesthesia monitoring during surgery.

## Data Availability

The libordpat library is available from https://github.com/seb-berger/libordpat under the terms of a BSD license.
